# In-Situ Temperature Measurement on CMOS Integrated Micro-Hotplates for Gas Sensing Devices

**DOI:** 10.3390/s19030672

**Published:** 2019-02-07

**Authors:** Marco Deluca, Robert Wimmer-Teubenbacher, Lisa Mitterhuber, Johanna Mader, Karl Rohracher, Marco Holzer, Anton Köck

**Affiliations:** 1 Materials Center Leoben Forschung GmbH, Roseggerstrasse 12, 8700 Leoben, Austria; robert.wimmer-teubenbacher@mcl.at (R.W.-T.); lisa.mitterhuber@mcl.at (L.M.); marco.holzer@mcl.at (M.H.); 2 ams AG, Tobelbader Strasse 30, 8141 Premstaetten, Austria; johanna.mader@ams.com (J.M.); karl.rohracher@ams.com (K.R.)

**Keywords:** silicon, MEMS, micro-hotplates, gas sensors, Raman spectroscopy

## Abstract

Metal oxide gas sensors generally need to be operated at elevated temperatures, up to and above 400 °C. Following the need for miniaturization of gas sensors and implementation into smart devices such as smartphones or wireless sensor nodes, recently complementary metal-oxide-semiconductor (CMOS) process-based micro electromechanical system (MEMS) platforms (micro-hotplates, µhps) have been developed to provide Joule heating of metal oxide sensing structures on the microscale. Heating precision and possible spatial temperature distributions over the µhp are key issues potentially affecting the performance of the overall gas sensor device. In this work, we use Raman spectroscopy to directly (in-situ and in-operando) measure the temperature of CMOS-based µhps during the application of electric current for Joule heating. By monitoring the position of the Raman mode of silicon and applying the theoretical framework of anharmonic phonon softening, we demonstrate that state-of-the-art µhps are able to reach the set temperature with an error below 10%, albeit with significant spatial temperature variations on the hotplate. This work demonstrates the potential of Raman spectroscopy for in-situ and in-operando temperature measurements on Si-based devices, an aspect of high relevance for micro- and nano-electronic device producers, opening new possibilities in process and device control.

## 1. Introduction

Metal oxide (MOX) based gas sensors have been developed over decades to established devices and represent the current state-of-the-art in chemical sensor technology. MOX sensors provide the opportunity for cheap and simple fabrication but require to be operated at high temperatures. This can be explained by the generally accepted ionosorption model, which describes the interaction of metal oxide materials with ambient gases mediated by adsorbed oxygen species on the MOX surface. Barsan et al. [1] have shown that the availability of oxygen species depends on the temperature, and that atomic oxygen is the dominant oxygen species for the gas sensing processes. This species is formed abundantly above 150 °C, and its amount further increases with temperature. Consequently, the optimal operation temperature range of MOX gas sensors is 250 °C to 400 °C, which results in the need of a heating system.

Early MOX gas sensors were heated by a macroscopic heating structure (heating filaments) [2,3]. Since the application for these gas sensors was the detection of flammable gases in household applications (cooking stove), power consumption was not a primary issue. One of the first steps to low power devices was made by Chang et al. [4] in 1986. The technique to fabricate a micro heating structure was inspired by piezoresistive/piezocapacitive pressure sensors. They integrated a SnO_2_ sensing element onto a thin silicon membrane containing a microscopic heater, i.e., a micro hotplate (µhp). These silicon membranes were fabricated by an anisotropic wet chemical etching method on silicon substrates. The maximum temperature achieved with these µhps was around 300 °C. A further advancement was the integration of tin dioxide on a CMOS fabricated µhp by Suehle et al. [5] with a maximum hotplate temperature of 500 °C. Hereby, the CMOS MEMS structures were fabricated at a commercial foundry. Semancik et al. [6] advanced the development of these µhps by applying an insulated metal layer above the heater to distribute the heat more evenly. In contrast to the work of Suehle et al.—where the heating element was a heating filament—Udrea et al. [7] designed a µhp heated by a metal-oxide-semiconductor field effect transistor (MOSFET) heater. The entire chip was fabricated in silicon-on-insulator (SOI)-CMOS technology, which allows higher operation temperatures and the integration of operation circuitry adjacent to the µhp structure [7]. SOI-CMOS based µhps can reach 500 °C with a power consumption of 30 mW, as reported by Ali et al. [8]. The integration of a µhp with operation circuitry on CMOS technology was done by Barrettino et al. [9]. These µhps have a power consumption of 35 mW when heated to 350 °C. In our group [10] we have already reported on a fully CMOS integrated µhp operated at 350 °C with a power consumption of roughly 12 mW in direct current (DC) operation. This approach enables the implementation of electronics and is the technology of choice for the development of smart gas sensor devices for consumer electronic applications. Power consumption can be further reduced by operating in a pulsed heater operation [11]. In addition to gas sensor applications, µhps are very promising as IR micro-emitters for spectroscopy applications, enabling the development of miniaturized IR spectrometers for gas detection [12].

Knowledge about the absolute temperature of such µhps is key for controlled and reproducible gas detection, as well as for controlled infrared (IR) emission. In addition, temperature distribution over the surface of the hotplate is a very important issue. Both absolute temperature measurement as well as temperature distribution can be addressed with IR thermography employing thermal imaging cameras [13]. This technology is based on the theory of black body radiation and basically enables the determination of absolute temperature in relation to the detected radiated heat. However, while this technology is very useful for bulk materials for measuring the absolute temperature, IR thermography cannot be directly applied on heated thin membranes, such as CMOS based µhps. Those structures are semitransparent in the IR regime, and do not exhibit black body radiation due to the different materials involved (Si, SiO_2_, metals etc.) and due to surface properties, such as different processing-induced roughness and topography. Thus, to make IR measurements possible, the µhps have to be coated with an additional material such as black paint coating mimicking black body radiation, which can strongly affect the thermal mass of the µhp and thus the measurement result. Moreover, this method requires very expensive IR lens setups to provide useful resolution of the small µhp (size 80 × 80 µm^2^). The spatial resolution over the µhp is strongly limited by the pixel size and the number of the IR camera, and is typically larger than 30 µm (depending on the detected wavelength). This often prevents the detection of small hot spots on the µhp, which might indicate strong local heating due to reliability problems. As a result, absolute temperature measurements of micro-devices such as the µhp remains a very critical issue.

Raman spectroscopy is a powerful alternative approach to directly (in-situ) measure the absolute temperature of CMOS-based µhps during the application of electric current for Joule heating (i.e., in-operando). Raman spectroscopy has been often taken into consideration for in-situ temperature measurements [14,15,16,17]. The most straightforward way to determine the temperature from Raman spectra is to measure the intensity ratio of Stokes to Anti-Stokes lines [16,18], which requires the spectrometer being equipped with a notch filter. This method, however, does not consider the temperature dependence of the optical constants of the sample, nor the changes in the optical gap with temperature [14]. Both aspects are expected to modify the Raman cross section/intensity ratio and thus lead to errors in the measured temperatures above ~180 °C [14]. Hence, methods based on the variation of the Raman line peak position and/or linewidth with temperature (which is due to anharmonic terms in the vibrational potential energy [19]) have been proposed and are preferred. Balkanski et al. [20] laid down the theoretical framework, which was subsequently used for temperature mapping in silicon and diamond [14,15,17,21]. In addition, simplified phenomenological approaches have been proposed [14,22].

In this work, we measured the temperature of CMOS-integrated µhps in-situ and in-operando using micro-Raman spectroscopy. First of all, we calibrated the material’s response by applying heat externally and measuring the temperature at the sample surface with a temperature sensor during Raman spectral acquisition, while monitoring the peak shift. The temperature increase due to laser heating was also taken into account in the calibration by calculations based on the steady state heat equation applied to a Gaussian laser beam. Following the calibration, the temperature was measured across a (initially virgin) hotplate during operation (i.e., application of electrical current to the hotplate) along two perpendicular line scans. This work highlights possible issues in the design and fabrication of µhps for MEMS applications, and underlines the role of Raman spectroscopy for localized in-situ/in-operando temperature analyses.

## 2. Materials and Methods

**Figure 1:**
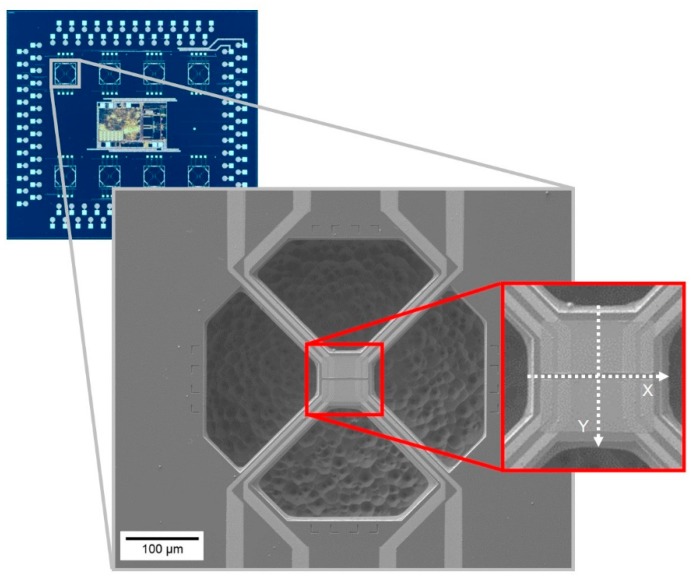
Fully processed CMOS chip with eight µhps, including magnification of a single µhp. The position and direction of the X and Y Raman line scans with respect to the µhp are indicated in the magnified view. Line scan X is carried out in the narrow gap perpendicular to the electrical contacts (visible as light grey color). In the displayed µhp a metal oxide layer was also deposited. This was not the case for the µhp measured in this work.

The CMOS chip used in this work was provided by ams AG (Figure 1) and contained eight µhps that could be operated individually. These µhps are MEMS structures consisting of thin thermally insulated membranes, which are solely connected to the adjacent chip by four narrow arms. The thermal insulation is achieved in a post processing step, where the membranes are subjected to a release etch resulting in the removal of the silicon in which the membranes are embedded prior to the etching process. The membrane itself contains an internal micro heater to set the temperature of the sensor. Additionally, a heat spreader was employed to ensure a uniform temperature distribution on the membrane surface. The wiring for the µhp heater was integrated into the four arms. The temperature of the µhp was measured by an integrated thermocouple, which was very useful for exact temperature control. However, temperature calibration of the thermocouple was difficult, because heating of the whole chip in well-defined temperature environment cannot be applied in this case. Electrical contacts to evaluate the electrical resistance of the gas sensor were processed on top of the µhp membrane. A four-wire setup was chosen for the resistance measurement. These electrical contacts were routed to the micro hotplates’ membrane on top of the four arms. In total, two sensors per hotplate could be measured in a four-wire configuration.

For the implementation of this temperature measurement method, two kinds of CMOS chips were prepared: A CMOS chip without the release etch and a fully processed CMOS chip with the release etch. The µhps on the CMOS chip without the release etch were used for calibration, and could not be operated via the internal heaters. Since these µhps were not thermally insulated, any heat generated by the internal heater would in fact be dissipated by the surrounding silicon. Therefore, an external platinum (Pt) heater (Delta R Pt 6.8) and two resistance temperature detector (RTD) sensors (Delta R Pt100) were attached to the chip to heat up the area of the µhps. Hereby, one RTD sensor and the heater were put below the chip, whereas the second sensor (which was used for comparison with the Raman measurements) was put on top of the chip. A thermal conductive adhesive (Aremco Ceramabond 865, Aremco Products Inc., Valley Cottage, NY, USA) was used to merge all the components. Heater and temperature sensors were operated by an external source. The fully processed CMOS chip, in which all µhps were released, was used for the final evaluation of the method. This CMOS chip was wire-bonded onto a printed circuit board (PCB), which allowed the operation of the internal heaters of each µhp separately.

Raman measurements were performed in a LabRAM HR800 spectrometer (Horiba Jobin Yvon, Villeneuve d’Ascq, France) using 514.5 nm excitation from an Ar-ion laser with 2.32 mW power on the focused spot on the sample. This power ensured a high signal-to-noise ratio of the Si peak without excessively heating the sample surface (cf. Section 3.2). The laser light was focused (and the scattered light collected) using a 100× long-working-distance objective lens (LMPLFLN, Olympus, Tokyo, Japan). The numerical aperture (NA) of 0.8 ensured a laser spot diameter smaller than 1 µm, while the penetration depth of the laser, defined as the material depth contributing to 95% of the intensity signal (measured according to Refs. [23,24]), was ~500 nm for the used experimental settings. The collected light was passed through a notch filter in order to remove the elastically scattered components, then it was dispersed in the single-stage spectrograph (focal length: 800 mm) using an 1800 gr/mm grating. A multichannel detector (Charge-Coupled Device, CCD) was used to visualize the spectrum. In order to compensate for thermal fluctuations of the equipment, in each measured point, the observed spectral shift was corrected by the 540.06 nm Ne emission line obtained from a Pen-Ray source (LOT-Quantumdesign, Darmstadt, Germany).

The calibration procedure was carried out as follows: First, the laser was focused at the center of one µhp in the calibration sample (the CMOS chip without the release etch). Then, the whole structure was heated up by the Pt heaters and the temperature was measured and controlled via the temperature sensor placed above the Si surface. Using the upper temperature sensor ensured more precision of calibration, since its location is closer to the area effectively measured by the Raman. The voltage was supplied to the Pt heaters using a source meter unit (SMU, Keithley 2401, Keithley Instruments, Cleveland, OH, USA), and the temperature sensor output was read-out using a digital multimeter (DMM, Keysight 34401a, Keysight Technologies, Santa Rosa, CA, USA) and converted to temperature using a calibration table provided by the sensor manufacturer (Delta R GmbH). There were 19 temperature steps carried out between room temperature and ~405 °C; at each temperature step, first of all, the voltage was adjusted in order to reach the desired set temperature. Then, a waiting time of 10 min was used to allow the whole system to reach the steady state. After that, the temperature (i.e., the temperature sensor output) was recorded and the Raman measurement started. Each spectrum required only 21 s to be measured, but a total of 4 points were measured per temperature step, in order to improve accuracy. Following the heating run, a few spectra were also collected during cooling (at ~350 °C, 270 °C, 195 °C, 70 °C, and 30 °C) in order to check the reproducibility.

Subsequently, the temperature measurement was performed on virgin, fully-processed µhps (not connected to any external heater) during the application of the electric current via the SMU. No external temperature sensor was used to control the temperature, but the desired temperature was set according to the supplied current and the internal thermocouple reference. Five temperature steps were carried out at 25 °C, 250 °C, 300 °C, 350 °C and 400 °C, which (above 250 °C) are relevant operating temperatures for metal oxide gas sensors. After setting each temperature, a waiting time of 10 min was used to allow it to reach steady state. The measurement consisted of two perpendicular line scans (each of ca. 22 points, i.e., a step size of ~3 µm), named X and Y, carried out at the center of the µhp (cf. Figure 1). The purpose of this measurement was to check the precision and stability of the µhps at different set temperatures, and verify the presence of hot or cool spots.

The collected Raman spectra were fitted using pseudo-Voigt profiles in the Labspec software environment (Horiba Jobin Yvon). Other calculations in the present work were carried out using Origin 9.1 (OriginLab, Northampton, MA, USA) and Scilab (Rungis, France) software.

## 3. Results and Discussion

### 3.1. Effect of Temperature on the Raman Spectrum of Silicon

**Figure 2:**
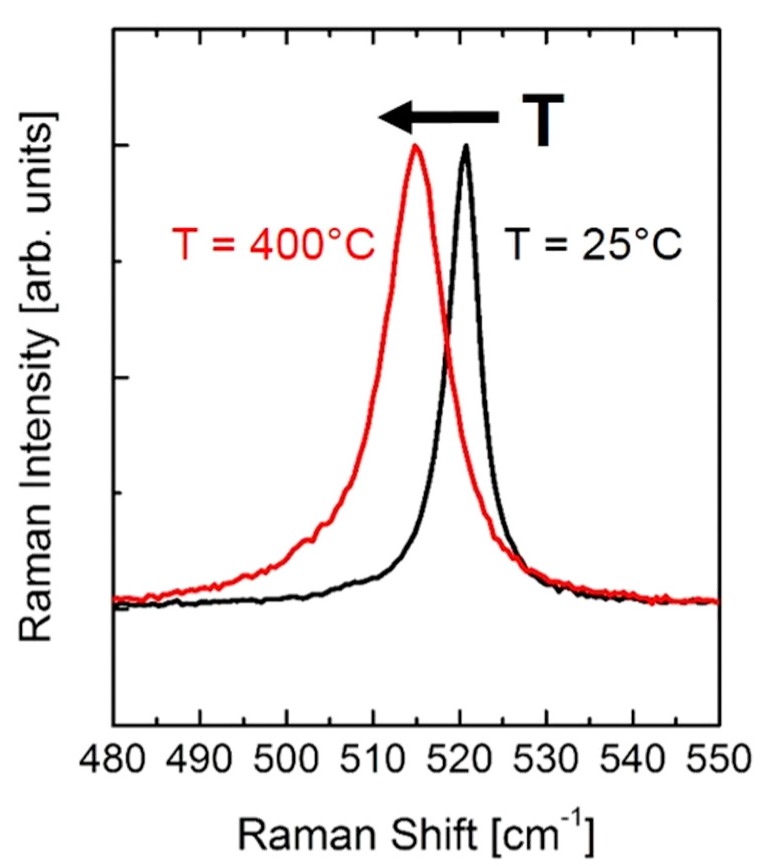
Raman spectrum of polycrystalline silicon at 25 °C and ~400 °C, as collected on the µhps. Increasing T produces a shift of the Raman line position and a broadening of the linewidth.

Figure 2 shows the normalized Raman spectrum of polycrystalline silicon as measured on the µhp samples at 25 °C and at ~400 °C. Clearly, increasing the temperature produces a shift of the Si peak position towards the lower Raman shift values, together with a broadening of the Si Raman linewidth.

Balkanski et al. [20] demonstrated that in order to reproduce the peak shift and linewidth (i.e., Full Width at Half Maximum, FWHM) in dependence of temperature, it is necessary to consider anharmonic terms up to four phonons, which gives the following relationships for peak shift and FWHM, respectively:

(1) $$\omega \left(T\right)=\omega_{0}+A\left(1+\frac{2}{e^{x}-1}\right)+B\left(1+\frac{3}{e^{y}-1}+\frac{3}{{\left(e^{y}-1\right)}^{2}}\right),$$

(2) $$\Gamma \left(T\right)=C\left(1+\frac{2}{e^{x}-1}\right)+D\left(1+\frac{3}{e^{y}-1}+\frac{3}{{\left(e^{y}-1\right)}^{2}}\right).$$

where $x=\hbar \omega_{0}/2k_{B}T$ and $y=\hbar \omega_{0}/3k_{B}T$. *ω*_0_ is the position of the Raman spectral line of Si at 0 K and is taken as the fitting parameter. In addition, *A*, *B*, *C*, and *D* are fitting parameters. *k_B_* is the Boltzmann’s constant, and ℏ is the reduced Planck’s constant.

Cui et al. [14] proposed an alternative formula to describe the temperature dependence of the Raman peak position:

(3) $$\omega \left(T\right)=\omega_{0}-\frac{E}{e^{F\frac{{hc\omega }_{0}}{k_{B}T}}-1}.$$

Where *h* is Planck’s constant, *c* the speed of light in vacuum, and *E* and *F* are fitting parameters. Equation (3) differs from Equations (1) and (2) derived by Balkanski et al., in the sense that it considers electron-phonon interaction resulting in the renormalization of the phonon frequencies, whereas Equations (1) and (2) consider only phonon–phonon interactions. In fact, Equation (3) was chosen to have the same functional form as that of the equation describing the renormalization by electron-phonon interaction of the band gap in the Einstein approximation [25]. Cui et al. provided a comparison of the two approaches in diamond, and concluded that Equation (3) leads to more precise fits of the phonon shift with temperature. Moreover, Equation (3) provides ease of calculation, since the temperature is obtained by simply inverting Equation (3):

(4) $$T=\frac{{Fhc\omega }_{0}}{k_{B}ln\left(1+\frac{E}{\omega_{0}-\omega \left(T\right)}\right)}.$$

Hence, in each measured point the temperature can be calculated directly from the obtained peak position, provided that the parameters *E*, *F*, and *ω*_0_ have all been previously determined with a suitable calibration procedure. In the following, we applied and discussed the suitability of both approaches by Balkanski et al. and Cui et al. to the calibration of the Raman response to temperature variations in the polysilicon-based µhps, considering also the temperature increase caused by focusing the laser on the surface of the polysilicon µhps.

### 3.2. Effect of Laser Heating on the Surface of Polysilicon µhps

**Figure 3:**
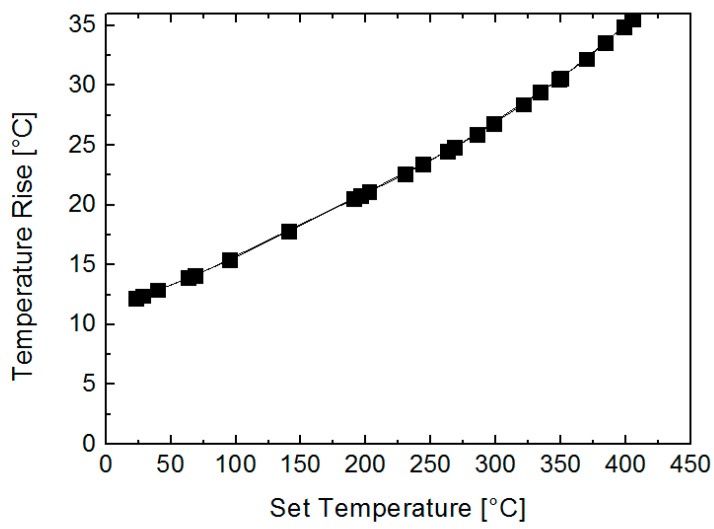
Temperature increase up to 400 °C caused by the laser heating on the surface of polysilicon at different temperatures. The induced temperature rise increases with the ambient temperature dominated by the thermal conductivity, which decreases with temperature.

The absorbance of the laser energy during the Raman measurements led to a temperature increase within the polysilicon µhps. The effect of laser heating was calculated by considering a semi-infinite polysilicon substrate [26]. The intensity of the laser was assumed to be Gaussian distributed and the laser energy input was treated as a surface heat source, as the measured optical penetration depth of 500 nm is small compared to the thermal diffusion length (>20 mm). The temperature rise on the surface was calculated using the steady state heat equation [27]:

(5) $$T\left(x,y\right)=\iint \;A\;\frac{8P}{{\pi d}^{2}}e^{-{\left(\frac{2\;\times \;\sqrt{2}}{d}\right)}^{2}\left(x^{2}+y^{2}\right)}\frac{1}{2\pi \kappa \left(\sqrt{x^{2}+y^{2}}\right)}dx\;dy.$$

where *A* is the absorbance of the laser, P the laser power and d the diameter of the laser spot. The material parameters of the polysilicon, like the thermal conductivity κ, but also *A*, are temperature-dependent [28], so the temperature increase was calculated for all the different temperatures set by the Pt-heater during calibration. In Figure 3, the Gaussian weighted average of the temperature rise can be seen as a function of each set temperature. The laser induced temperature rise increases with increasing temperature due to the temperature-dependent material parameters of the polysilicon.

### 3.3. Calibration of Phonon Response with Temperature in µhps

**Figure 4:**
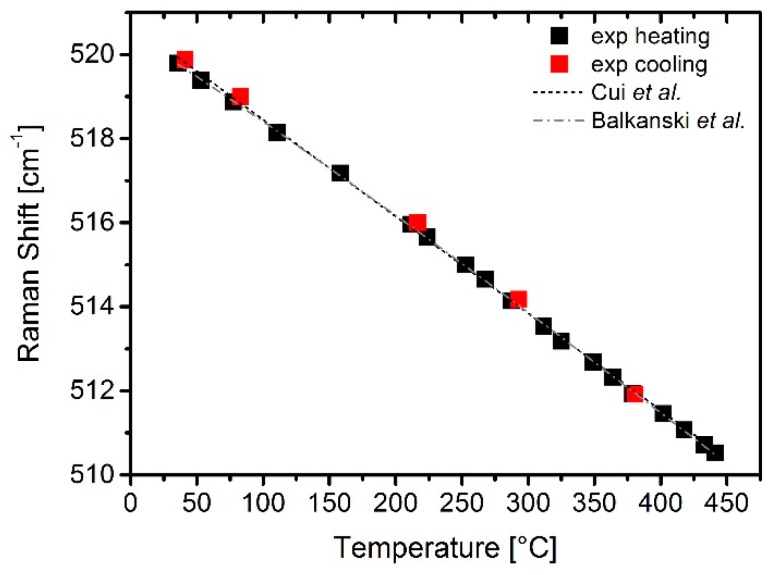
Calibration (heating/cooling) of peak position with fitting curves.

The results of the calibration measurement with an external controlled temperature increase on the µhps is presented in Figure 4 with a fitting curve provided by Balkanski’s (Equation (1)) or Cui’s (Equation (3)) equations. The measured peak shift is plotted against temperature values corrected by the laser-induced heating temperature at each temperature step set by the Pt heater, according to the values displayed in Figure 3. Cooling down after completion of the heating cycle allowed us to obtain similar values of the measured peak position, proving that the phonon shift with temperature is a reversible process. Best-fitting the experimental values collected on heating, the following values of the fit parameters were obtained for Balkanski’s equation: A = −4.174; B = −0.043; ω_0_ = 527.62 cm^−1^; for Cui’s equations the best-fitting values were E = 0.035; F = 0.002; ω_0_ = 527.04 cm^−1^. The better fit of Balkanski’s equation in the temperature range up to ~150 °C (cooling points were not used in the fit) suggests that in this range electron-phonon interactions are not relevant and the Raman peak shift with temperature can be described with phonon–phonon interactions alone. Thus, temperature calculation from the measured peak shift has been performed in this paper only with Balkanski’s equation, although for higher temperatures Cui’s equation would likely perform similarly well. The choice of restricting the analysis only to the peak position is motivated by the fact that the error on the FWHM (not shown) was as high as ±30 °C due to the higher sensitivity to fitting errors. The error on the determination of the peak position, on the other hand, was estimated as ±4 °C.

### 3.4. In-Situ Temperature Measurements on µhps

**Figure 5:**
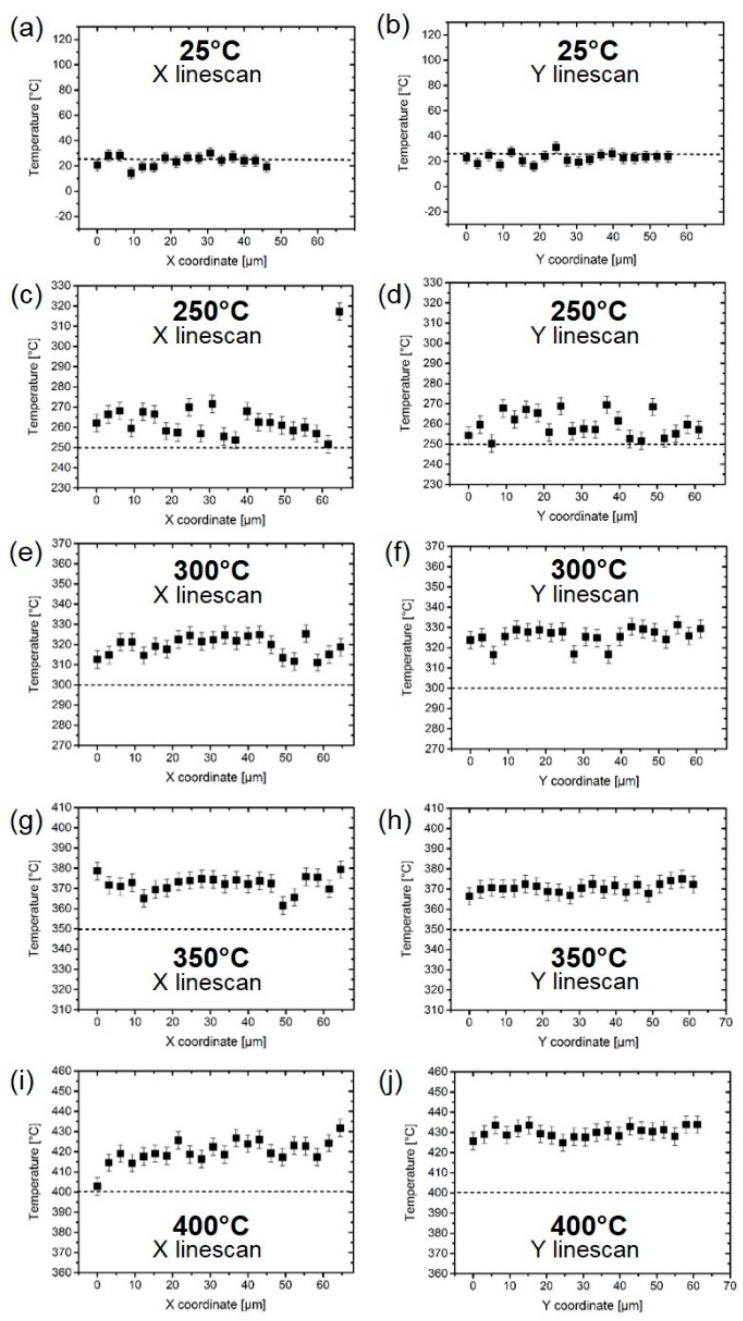
Temperatures measured on the surface of Si µhps at different set temperatures and in X and Y directions. (**a**,**b**) 25 °C, X and Y; (**c**,**d**) 250 °C, X and Y; (**e**,**f**), 300 °C, X and Y; (**g**,**h**) 350 °C, X and Y; (**i**,**j**), 400 °C, X and Y. The line scans at 25 °C were interrupted earlier. Note the increasing discrepancy between set and measured temperatures for increasing T.

Figure 5 displays the results of line scans performed in the X and Y directions across the µhps at different set temperatures (25 °C, 250 °C, 300 °C, 350 °C, and 400 °C). The temperature was calculated from the measured peak shift using Equation (1) according to the calibration displayed in Figure 4. Since the temperature calculated this way is still influenced by laser heating, in order to obtain the true temperature of the micro-hotplate, the laser heating temperature (calculated as described in Section 3.2 and considering the thermal properties of Si at the used set temperatures) was subtracted from the value obtained from Equation (1). The temperature values displayed in Figure 5 are thus representative of the thermal response of the µhps at the different set temperatures. At 25 °C the hotplate is able to maintain, on average, the set value, but for higher temperatures a discrepancy between the set value and the measured temperature on the hotplate is evident. Moreover, the X and Y line scans evidence the presence of hot and cool spots throughout the micro-hotplate. Although some of these fluctuations could be due to statistical dispersions, some trends can be recognized, especially in the neighborhood of electrodes. This effect seems evident particularly on the X line scans, which were carried out in the narrow gap between electrodes, visible in Figure 1. Comparing Figure 5e,g (i.e., X line scans at 300 °C and 350 °C, respectively), for instance, the presence of symmetrical cooler spots (with reference to the line scan center) corresponding to the locations in proximity of the electrodes can be noted. Further analyses (i.e., multiple line scans) will be performed in the future to rule out the effect of statistical dispersions.

**Figure 6:**
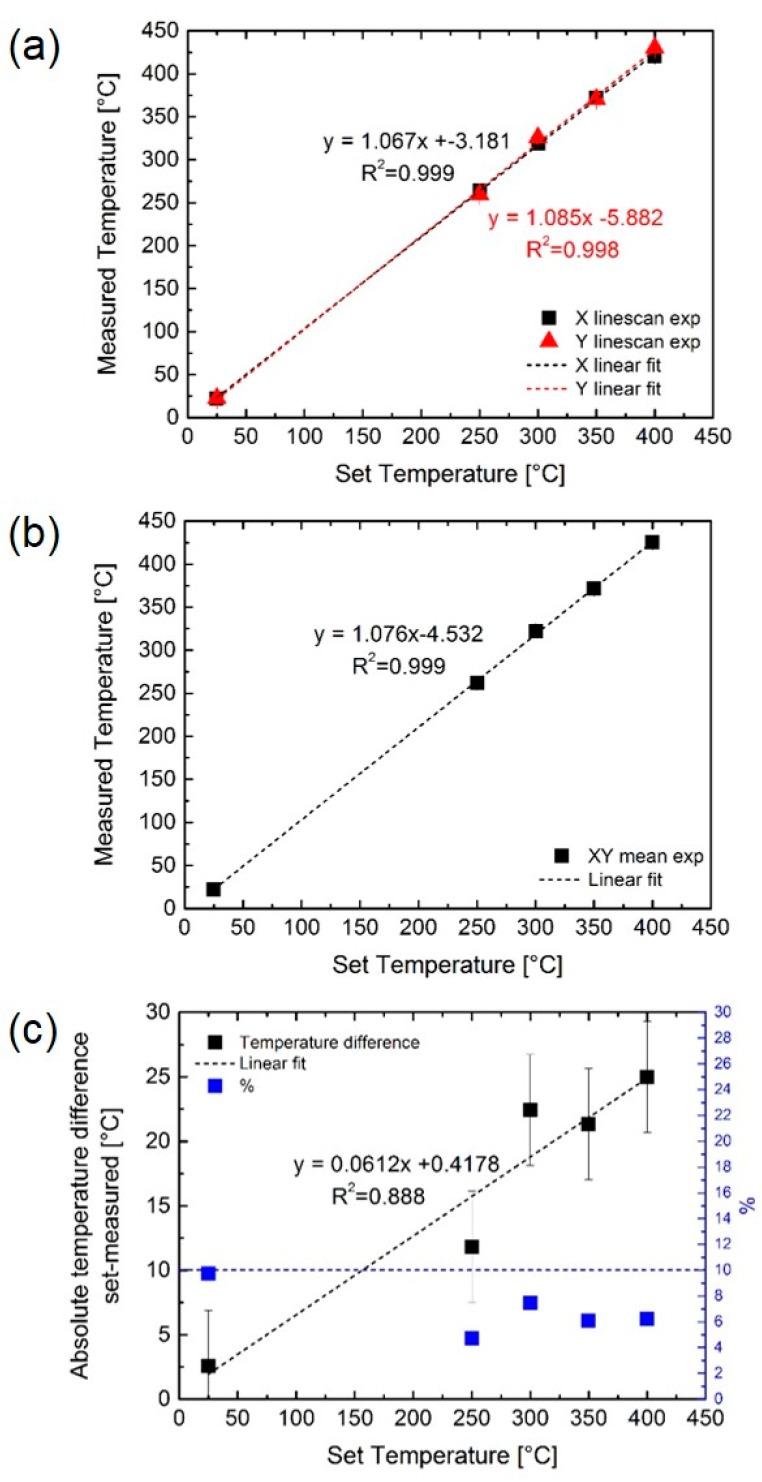
(**a**,**b**) Difference between the set and measured temperatures on the Si-µhp: X-Y line scans and the average between the line scans, respectively. (**c**) Absolute temperature difference between set and measured temperatures, including the error percentage (blue symbols—right y-axis). The dashed horizontal blue line symbolizes the 10% error threshold. The error remains below this threshold at all measured temperatures.

The difference between set and measured temperatures is displayed in Figure 6a for both the X and Y line scans, whereas Figure 6b shows the average of both line scans for all temperatures. As can clearly be seen, the measured temperatures slightly overestimate the set temperature, and the discrepancy (already evident from Figure 5) increases with increasing set temperature, being ~12 °C at 250 °C and ~25 °C at 400 °C (cf. Figure 6c). Despite this difference, the error always remains below 10%, as seen in Figure 6c by the blue symbols. The highest error percentage is at 25 °C, probably due to the higher influence of the ambient temperature at a low µhp heating power. The discrepancy between the set and measured temperature was likely to be ascribed to the construction of the hotplates, in which the embedded thermocouple was not located on the heater structure but on two of the four connecting arms (cf. Figure 1).

All in all, it can be argued that the measured µhps are able to reach the set temperatures in operation reliably and within 10% error. This error is systematic and could be corrected by an experimental calibration curve, thereby allowing the µhp to be heated to the optimum working temperature. The presence of hot/cool spots on the µhp surface (whose presence was suggested by this work), however, if combined with inhomogeneous metal oxide distribution on the µhp surface might be a critical issue that complicates the interpretation of the gas sensing results.

## 4. Conclusions

In this work, we carried out a thorough evaluation of the temperature generated by electrically-driven CMOS-integrated µhps, by measuring the temperature during operation of the µhps by Raman spectroscopy. First, a calibration of the Raman spectroscopic response was carried out, also considering the temperature increase caused by the laser heating. Then, we measured the temperature on the hotplates by performing line scans in the X and Y directions, that is, perpendicular or parallel to the electrodes on the hotplate, respectively. Our study showed that µhps can reach the set temperature with an error below 10% but amounting to up to a ~25 °C difference at a 400 °C set temperature. We also suggested the presence of a hot/cool spot on the surface of the µhps. These issues may affect gas sensing measurements to a great extent, and thus call for a proper redesign (e.g., thermocouple location) or calibration of the CMOS integrated micro-hotplates to enable more precise temperature control. This work demonstrates the potential of Raman spectroscopy for in-situ and in-operando absolute temperature measurements on Si-based devices. Raman spectroscopy provides a high spatial resolution and might be the technology of choice for temperature measurement of micro- and nano-electronic devices. This is of high relevance for microelectronic device producers, opening new possibilities in device characterization and device control.
